# Sirolimus Ointment for Facial Angiofibromas in Individuals with Tuberous Sclerosis Complex 

**DOI:** 10.1155/2017/8404378

**Published:** 2017-11-15

**Authors:** S. Amin, A. Lux, A. Khan, F. O'Callaghan

**Affiliations:** ^1^Paediatric Neurology, University College London and University Hospitals Bristol, London, UK; ^2^Paediatric Neurology, University Hospitals Bristol, Bristol, UK; ^3^Dermatology, University Hospital of North Durham, Durham, UK; ^4^Paediatric Neurosciences, University College London, London, UK

## Abstract

*Background. *Facial angiofibromas affect most patients with tuberous sclerosis complex. They tend to progress, can cause recurrent bleeding and facial disfigurement, and have significant psychological effects. We reviewed the effectiveness and safety of topical sirolimus ointment 0.1%. We also assessed the effect of treatment on quality of life.* Methods.* We report our experience in using sirolimus ointment in 14 patients with TSC (9 children and 5 adults). The impact of sirolimus ointment was monitored with digital photography, dermatological review using a validated Facial Angiofibroma Severity Index (FASI), and quality of life assessments using the questionnaires PedsQL for children and SF36 for adults.* Results. *The FASI scores were improved in 12/14 cases after six months' treatment, and improvement was more likely in children (median FASI scores of improvement after treatment were 3 points for children and 1 for adults). Proxy-reported PedsQL scores for the total psychosocial domain improved significantly in the children in the cohort with treatment.* Conclusions*. Sirolimus ointment 0.1% administered once a day was effective in treating facial angiofibromas. It appears to be safe and well tolerated and to have a positive impact on patients' quality of life. It appeared to be most beneficial when started in childhood.

## 1. Introduction 

Tuberous sclerosis complex (TSC) is a neurocutaneous condition which has an autosomal dominant pattern of inheritance. Approximately two-thirds of cases are sporadic. The birth incidence has been estimated as 1 in 5,800 per year [1].

This condition is caused by mutations in the tumour suppressor genes* TSC1 *and* TSC2*.* TSC1* encodes for the protein hamartin which is located on chromosome 9, and* TSC2* encodes for the protein tuberin, located on chromosome 16 [2, 3]. Hamartin and tuberin function together within the cell as a complex and have an inhibitory effect on the mammalian Target Of Rapamycin (mTOR), a protein kinase that affects cell growth and division through the regulation of protein synthesis [4, 5]. Mutation in either* TSC1 *or* TSC2* genes leads to overactivation of mTOR pathway, which will lead to uncontrolled cell growth. This, in turn, causes growth of benign tumours (hamartomas) in multiple organs such as the brain, kidneys, skin, heart, and lungs, which are the clinical hallmarks of the disease.

Approximately 86% of patients with TSC suffer from facial angiofibromas, which are benign tumours on the face. They generally become noticeable at around the age of five years. The severity varies significantly between patients. They can have a huge impact on a patient's self-esteem [6]. In addition, they are known to cause recurrent bleeding, irritation, infection, facial scarring, and disfigurement [7]. The mTOR inhibitors mimic the action of the* TSC* gene products, by inhibiting the action of mTOR and therefore regulating the PI3 kinase-mTOR-S6 kinase intracellular growth pathway. Sirolimus (also known as rapamycin®) and its 40-*O*-(2-hydroxyethyl) derivative, everolimus, effectively inhibit mTOR [8–10]. In recent years, systemic mTOR inhibitors have been used to treat the complications of TSC and it has been shown that they shrink TSC lesions such as renal angiomyolipomas and subependymal giant cell astrocytomas and that they improve facial angiofibromas [11, 12]. However, their use is constrained by concerns about systemic side effects. There have been several case reports of sirolimus ointment for the treatment of facial angiofibromas, where different sirolimus ointment strength, preparation, and regimes have been used. None of these studies have used a validated tool to assess the severity of the rash and its response to treatment [13].

The best-established treatment options to date for facial angiofibromas are laser therapy and surgical removal of the large lesions. However, many patients also have learning difficulties and do not tolerate the sessions of laser therapy without multiple general anaesthetics. We report our experience in using sirolimus ointment 0.1% for facial angiofibromas in children and adults with TSC, using a standardised and validated Facial Angiofibroma Severity Index. We also assessed the effect of this treatment on quality of life.

## 2. Materials and Methods

Fourteen sequential patients with a definite diagnosis of TSC, as defined by the International Tuberous Sclerosis Complex Consensus Group [14], from our TS clinic at the Royal United Hospital in Bath started using sirolimus ointment from May 2014. Any patients who were suitable were offered the treatment. We had 14 patients suitable for this treatment at the time. We did not exclude any patients. Any patients who attended the clinic in May 2014 and had facial angiofibromas agreed to try sirolimus ointment. None of the patients were on systemic mTOR inhibitors before starting or during this treatment of topical sirolimus.

The gender balance and prevalence of learning disabilities in our clinic cohort are similar to previously reported population based TSC cohorts, suggesting that this clinic population is not grossly dissimilar from the TSC population at large [15]

Topical 0.1% sirolimus ointment was used. The patients were advised to apply a thin coating to the affected areas on the face, once a day in the evening. Each ointment pot contained 30 mg of sirolimus in 30 grams of ointment, and this consisted of 15 tablets of sirolimus (rapamycin 2 mg tablets) crushed and mixed with white soft paraffin.

One pot was given for 6 weeks. Each pot costed approximately £180. The treatment was not funded by any pharmaceutical companies. The cost was covered by the NHS as this treatment was part of patient's standard care. An information leaflet about topical sirolimus therapy was given to our patients at the start of the therapy. Patients were advised to use hydrocortisone cream should they suffer from irritation or a burning sensation. We routinely use digital photography to monitor the effectiveness of topical sirolimus in our patients. Digital photography was undertaken at baseline, and then at six months. Six photographs were taken by a digital camera from each patient at each visit. Three photographs were taken with the camera flash on and the other three are taken with the flash in automatic mode. One photograph was taken in full face view directly facing the camera and the other two were side profiles. The preferred facial expression was natural with both eyes open. However, a facial smile was acceptable. Patients were advised not to wear makeup before having their photo taken. None of the patients in this series wore make up during the visits.

During each visit, patients, parents, and carers were asked to report on the degree of improvement. The treating physicians were also reporting the effect of the ointment. In order to obtain a more objective assessment of the treatment our consultant dermatologist analysed the photographs and scored treatment response in a blinded fashion. The dermatologist was given the pre- and posttreatment photos for each patient without knowing whether they were pre- or posttreatment photos. The Facial Angiofibroma Severity Index (FASI) was used by the dermatologist to assess the severity of the rash.

The FASI is an objective clinical tool that assesses the severity of facial angiofibromas and treatment response. The index has good interrater reliability (correlation coefficient *s* > 0.98; range 0.97–0.99) [16]. The score is obtained by summing the partial scores assigned to each of three features: erythema, size, and extent of lesions. Erythema and lesion size are scored from 0 to 3, while the extent is scored 2 to 3. Mild, moderate, and severe facial angiofibroma correspond with activity score ranges on the FASI of ≤5, 6-7, and ≥8, respectively (Table 1).

We use the Pediatric Quality of Life Inventory (PedsQL) in our clinic to monitor children's quality of life. PedsQL is a reliable and valid assessment tool for quality of life in children. It is also concise, has age appropriate versions, and has parallel forms for children and parents [17]. For the adult patients, we use the Short Form 36 Health Survey Questionnaire (SF-36) to assess the quality of life [18]. FASI scores pre- and posttreatment were compared using Wilcoxon signed-rank sum test. Mann–Whitney* U* Test was used to compare the FASI scores between children and adults. Quality of life scores were compared using Student's paired* t*-test. Ethical approval was not required as this treatment is being used as part of patient's routine care. This is not a clinical trial. This treatment was available to all patients who were suitable. Consent was taken from each patient for taking facial photographs.

## 3. Results

### 3.1. Patient Characteristics

Fourteen patients (9 children and 5 adults; 7 male) were in this cohort. The median age was 16 and the range was from 9 to 40 years. Five cases had learning difficulties. Six cases had already received laser therapy or surgery for their facial rash in the past but significant angiofibromatosis remained. None of these patients received laser therapy or surgery in the 6 months prior to starting or during the topical treatment. None of the patients in this group had received topical mTOR inhibitors before starting the topical sirolimus therapy and none of them had received systemic mTOR inhibitors before or during the course of the study. Before the treatment, 4 patients had the maximum FASI score of 9, 7 patients scored 8, and 3 had a FASI score of 7 (Table 1).

### 3.2. Treatment Response

**Figure 1:**
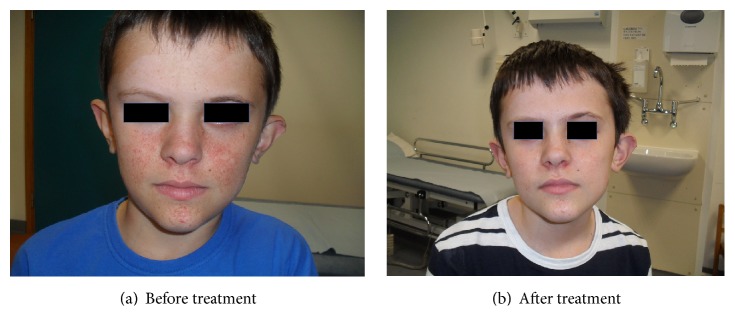


Based on the FASI photographic assessments by our dermatologist, FASI scores were improved in twelve out of fourteen (Figures 1 and 4 and Table 1) (2-tailed Wilcoxon signed-rank test *p* = 0.002). Of the remaining 2 patients, 1 had improvement in rash but no FASI score change. One had no detectable response. The treating physicians reported improvement in 13 out of 14 patients. Thirteen out of fourteen patients and their parents or carers reported facial angiofibroma improvement.

No one had worsening of facial angiofibromas after the therapy. Children (age 0–16 years) in this cohort responded better to the treatment than the adults. Median FASI scores improvement after treatment were three points for children and one point for adults (Mann–Whitney* U* Test *p* = 0.053). All the children had an improvement in their facial angiofibromas after 6 months compared with four out of five adults. The presence of learning disabilities had no apparent relationship with response to treatment. The median FASI scores improvement after treatment were 2 points for both patients with and without learning disabilities. Both female and male patients were equally responsive to this treatment.

### 3.3. Quality of Life

**Figure 2:**
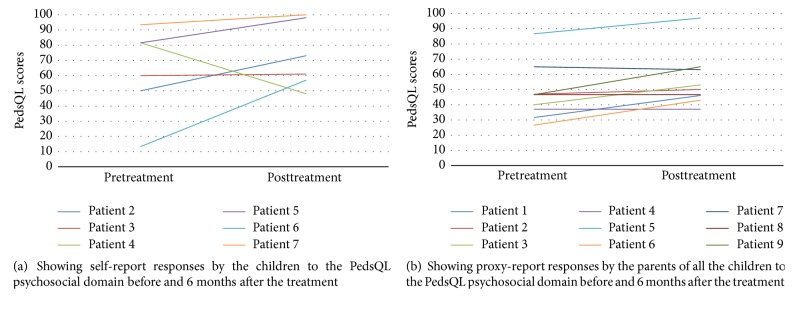


PedsQL was used in nine patients to assess their quality of life. Six children had no learning difficulties and completed the self-reported PedsQL. The self-reported scores for total psychosocial domain improved in five out of six patients (Figure 2(a)). The parents of all the nine paediatric patients completed the proxy PedsQL. The proxy-reported scores for total psychosocial domain improved significantly after treatment (paired *t*-test: *t* = −3.09, *p* = 0.014), with individual scores improving in five patients, staying the same in two and marginally declining in two (Figure 2(b)).

**Figure 3:**
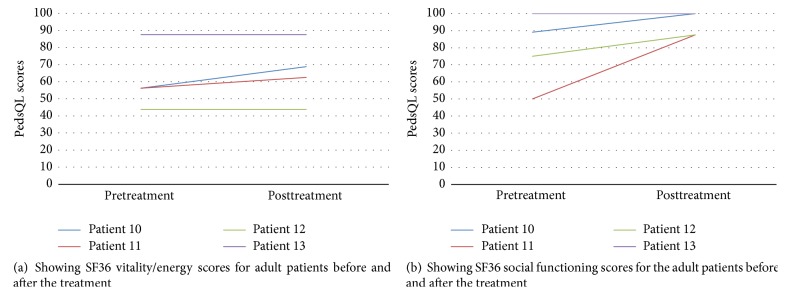


**Figure 4:**
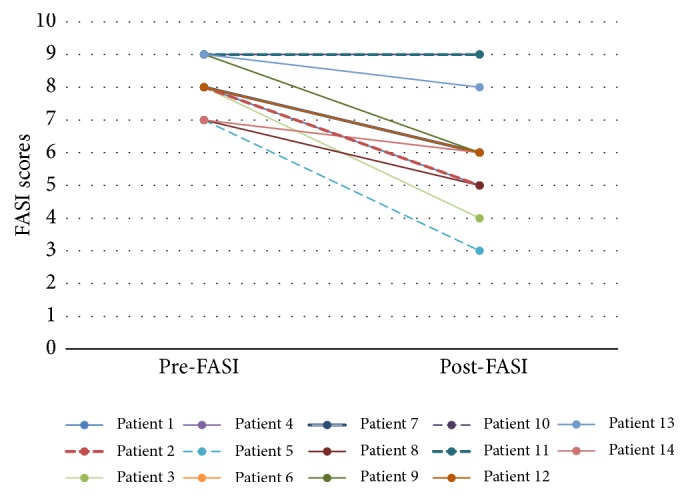
Showing FASI scores before and after treatment.

Four adult patients had their quality of life assessed using SF36. The vitality/energy domain scores of SF36 improved markedly in two patients and stayed the same in two (Figure 3(a)). The social functioning domain scores improved in three patients and declined in one (Figure 3(b)).

For the patient who had an unchanged FASI score but still reported rash improvement and also reported improvements in quality of life, his social function domain scores improved from 50 to 100 and vitality/energy from 56 to 69. The patient who had neither FASI nor facial angiofibroma improvement also had no improvement in quality of life.

### 3.4. Adverse Reactions

One patient developed facial redness two weeks after commencing the treatment. Hydrocortisone cream was used successfully to treat the reaction. He continued with sirolimus ointment and had no further reaction. No other patients reported any adverse events.

## 4. Discussion

This report shows that the facial angiofibromas, assessed by a dermatology assessment of photographs, using FASI, showed improvement in 12/14 TSC cases after 6 months of sirolimus ointment 0.1% treatment. The strength of this study is that it has attempted to objectively assess response of facial angiofibromatosis to topical mTOR inhibition using a validated instrument and an assessor blinded to treatment status. It has also attempted to describe the impact of treatment on the quality of life of patients. Sirolimus ointment and cream have been used in smaller case series. However, none of these reports have been from the UK and none have objectively assessed effectiveness and/or quality of life [19, 20].

One of the patients who did not show FASI score improvement had a very severe rash before treatment and it remained very severe after treatment. His rash had improved but not enough to change the FASI score. It may be that the FASI score is not sensitive enough to pick up improvement in patients with very severe rash. In particular, this patient's rash was less erythematous after treatment. Erythema is an important component of facial angiofibromatosis and it is the component that demonstrated the greatest improvement in the FASI scores. The degree of erythema noticed on visual inspection is reliably correlated with blood flow detected by Doppler scans [21]. It is probable that the topical mTOR inhibitor has been especially successful in reducing the vascularity of the angiofibromatosis. It is known that systemic mTOR inhibitors have a potent effect on the vascularity of other TSC lesions such as renal angiomyolipomas.

The better response amongst children compared to adults with this treatment is possibly due to the fact that the children's rash was less severe before the treatment compared with the adults or because the rash is still developing and therefore is more susceptible to intervention. We know from clinical experience that facial angiofibromas tend to stabilise in adulthood. The median FASI scores for children improved from 8 to 5.5 while for adults the scores were from 9 to 8. These results suggest that early intervention may be more effective and therefore justifiable.

The treatment has improved the quality of life of the children and adults in this study. The treatment specifically showed a significant impact on the psychosocial domain component of the quality of life scores. The psychological impact of facial angiofibromas on TSC patients may be underappreciated by healthcare professionals. In our experience, a lot of young children with TSC, who are mildly affected by the facial rash, still get teased or picked on at school by their peers. This can have a significant bearing on their school progress, which can also be associated with numerous physical, mental, and social harms [22]. The physical domains of quality of life were also assessed as they are part of the PedsQL and SF36 questionnaires. However, there was no difference in the physical domains before and after treatment in these patients. The parents and carers of one adult patient in this study have not completed an SF36 form on behalf of the patient as they found the questions difficult to answer. SF36 is a reliable tool and has content validity [18]. However, it may not be so reliable for adults with learning difficulties. There is no standardised and validated tool for quality of life assessment in adults with severe learning difficulties.

There are limitations with this case series report. Firstly, this is not a randomised placebo controlled trial and therefore the results may be subject to bias and there is certainly likely to be a lack of power in any statistical analyses. Secondly, we have not systematically measured compliance to treatment and therefore we cannot say that lack of response to treatment is not due to lack of compliance. However, it is reasonable to suggest that the improvement in facial angiofibromatosis demonstrated in this series is likely to be due to the topical treatment because we know from clinical experience that facial angiofibromas generally do not improve without intervention. Another limitation to the study is that the quality of life questionnaires for patients with severe learning difficulties was completed by parents and carers. This method of quality of life assessment is less reliable than self-reported outcome, but it is the only practicable method of assessment in people with learning disability, and it remains of interest. It is possible that the improvement we have seen in quality of life is due to a placebo effect or close follow-up. However, there is good correlation between response of rash to treatment and improvement in quality of life. All the patients who had facial angiofibroma improvement also had improvement in quality of life. In addition, we have seen more improvement in psychosocial domains rather than physical domains after the treatment.

All of the patients treated with this ointment have requested to continue the treatment and this high retention rate further suggests that it is an effective intervention with limited inconvenience or adverse effects. It will be interesting to see whether long-term treatment results in continued improvement. We do know from clinical experience that stopping treatment results in recurrence. We will continue to review them regularly as this treatment constitutes part of their ongoing clinical care. Unfortunately, not many patients in the UK have access to this ointment due to funding. We are hoping that reporting our experience of sirolimus ointment use will support family groups in streamlining funding for this treatment.

## 5. Conclusion

Sirolimus ointment 0.1% once a day was apparently effective in treating facial angiofibromas in our clinical cohort. It also appeared to be safe and well tolerated and had a positive significant impact on patients' quality of life. We saw greater effect in children than adults and therefore early treatment may be advisable. The positive outcomes from this case series suggest that a larger prospective placebo controlled randomised controlled trial is justified. The safety and efficacy of 0.1% sirolimus in this group set a baseline platform for a larger national study to compare 0.1% strength with a higher strength and placebo. There is a need for a national study to determine optimal dosing regimen of topical sirolimus for facial angiofibromas in TSC, to facilitate EMA licence application for this indication, and to facilitate NHS funding for this treatment. Currently, this treatment is not available through the NHS in all the Tuberous Sclerosis Clinics in the United Kingdom.

## Figures and Tables

**Table 1:** Showing patients characteristics and Facial Angiofibroma Severity Index (FASI) score before and after treatment.

Patient number	Age	Sex	Learning disabilities Present	Pre-erythema (0/1/2/3)	Pre-size (1/2/3)	Pre-extension (2/3)	Pre-FASI	Post-erythema (0/1/2/3)	Post-size (1/2/3)	Post-extension (2/3)	Post-FASI
1	12	♀	Yes	2	3	3	**8**	0	2	3	**5**
2	11	**♂**	No	2	3	3	**8**	1	2	2	**5**
3	13	**♂**	No	2	3	3	**8**	0	1	3	**4**
4	14	♀	No	2	3	3	**8**	1	2	3	**6**
5	16	**♂**	No	2	2	3	**7**	0	1	2	**3**
6	9	♀	No	2	3	3	**8**	1	2	3	**6**
7	17	♀	No	2	3	3	**8**	1	2	3	**6**
8	14	♀	Yes	2	2	3	**7**	1	2	2	**5**
9	9	**♂**	Yes	3	3	3	**9**	2	2	2	**6**
10	23	**♂**	No	3	3	3	**9**	3	3	3	**9**
11	27	**♂**	No	3	3	3	**9**	3	3	3	**9**
12	24	♀	No	2	3	3	**8**	1	2	3	**6**
13	22	**♂**	Yes	3	3	3	**9**	2	3	3	**8**
14	40	♀	Yes	1	3	3	**7**	0	3	3	**6**

*Erythema*. Skin color: 0, light red: 1, red: 2, and dark red/purple: 3. *Size*. Small (<5 mm): 1, large (>5 mm): 2, and confluent: 3. *Extension*. <50% cheek area: 2 and >50% cheek area: 3. *FASI Score*. < or = 5: mild, 6-7: moderate, and = or >8: severe.
